# Hospital and Institutional News

**Published:** 1917-09-29

**Authors:** 


### HOSPITAL AND INSTITUTIONAL NEWS.

### MEDICAL V.C.'S.

The posthumous award of a bar to the Victoria Cross conferred nearly a year ago on Captain Noel Godfrey Chavasse, R.A.M.C., son of the Bishop of Liverpool, is the highest tribute which it is possible to pay to the superlative heroism shown by a British medical officer at the Front. The magnificent devotion to duty and the self-sacrificing bravery of the doctors provide one of the most glorious pages in the annals of the great war. To win the V.C. twice indicates valour almost unparalleled in its splendour—indeed, Capt. Chavasse's case is, we believe, only the second since the distinction was first inaugurated by Queen Victoria in which such an award has been made. It is a matter of pride to the R.A.M.C. that the only other instance of a double V.C. is also provided by that corps, Lieut. (now Lieut.-Colonel) Arthur Martin-Leake, who had won the V.C. thirteen years before in South Africa, gaining the honour a second time about six months after the first shots had been fired in France. Though Lieut.-Colonel Martin-Leake was the first man to gain two V.C.s, Captain Chavasse was the first to win the honour twice in the same campaign. The record of the R.A.M.C. in regard to V.C.s during the present war is unsurpassed by any other section of the British Forces. Eight such decorations have been conferred, but the majority of the recipients gave their lives in performing the deed which honoured their name for ever. The surviving R.A.M.C. V.C.s, in addition to Lieut.-Colonel Martin-Leake, are Captain William Barnsley Allen and Captain George Allan Maling.

### LEGACIES FOR RATE-AIDED INSTITUTIONS.

It rarely falls to the lot of a rate-aided institution to have a legacy left by some kindly disposed person for the better provision of the inmates, but such is the experience of the Metropolitan Asylums Board. In 1912 Mrs. Elizabeth Rebecca Johnson died leaving £4,000 to the Southern Hospital, Dartford; £2,000 to the Northern Hospital, Winchmore Hill; and £1,000 to the North Eastern Hospital, Tottenham; whilst in the same year Mrs. Amy Charlton, of Manchester, bequeathed £200 to the Grove Hospital, Tooting. Naturally, the managers, who have the unlimited funds of the ratepayers to depend upon, were somewhat at a loss to know what to do with the gifts, but eventually, with the consent of the Local Government Board, they approached the Charity Commissioners, who consented to draft a scheme for the administration of the funds, after consultation with the medical superintendents of the hospitals. In 1914 the Charity Commissioners directed that the money should be invested by the managers as trustees, and it was enacted that after paying the expenses of administration the yearly income of each charity was to be applied "in, or towards, the cost of providing means of recreation calculated to increase the happiness and the general welfare of all those whose illness or employment leads to their being occupants of the hospitals to which the endowment of the charities is attached." The interest has accumulated, so that the capital of the two charities now stands at £10,262 invested in London 2½ per cent. Debenture Stock, and £800 in War Loan, producing an annual interest of £256 10s. 10d. and £40 respectively. The principal will remain intact, but the interest is devoted to providing such extras as, however desirable, cannot properly be supported out of the rates.

### FURTHER DISTRIBUTIONS FROM THE SALOMON ESTATE.

It will be remembered that the trustees of the estate of the late Mr. Leopold Salomon found themselves able to allocate a sum of £120,000 to charities as directed in the will. £30,000 of this amount was set aside for blind institutions and ophthalmic hospitals (see The Hospital, August 25, 1917), and a further distribution of £50,000 to hospitals has been announced. The London comes off best with a handsome grant of £5,000; Middlesex, Guy's, King's College, University College, the Royal Free, St. Bartholomew's, St. George's, St. Mary's, St. Thomas's, and Westminster Hospitals receive £2,000 each; and Charing Cross, the Seamen's, the Great Northern Central, the West London, the Cancer, Brompton, the National Hospital for the Paralysed, the London Lock, the Hospital for Sick Children, and the Royal Surrey (Guildford) are awarded each £1,000. This list accounts for £35,000, and the balance is given in sums of £500 and £250 to Metropolitan hospitals and one or two Surrey nursing associations. (Mr. Salomon was prominently associated with Surrey, having given Box Hill to the nation.) There remains a sum of £40,000 to distribute; the trustees have evidently considered the needs of the hospitals with great care and generosity, and the balance in their hands will probably be shared amongst miscellaneous charities.

### FORTY-NINE YEARS AS A GOVERNOR.

Mr. James Hora, a vice-president and joint treasurer of Westminster Hospital, who had been a governor for forty-nine years, has died after a life of generous work for the institution. His principal contributions included the gift of some £8,000 for the endowment of the "Marie Celeste" Ward for Children.

### A BIG WAR-TIME EXTENSION.

The extensive additions which have been thrown open at the Portsmouth Infectious Diseases Hospital deserve attention, for they are among the few new buildings which the Local Government Board thought sufficiently important to sanction. The Board's standard is one bed for every thousand persons, and the Portsmouth Infectious Diseases Hospital, with its 260 odd beds, even now only brings the hospital accommodation of the town up to this ratio. There is no provision for the future except a site at the back of the hospital, which has been purchased to allow for future expansion. The new buildings include two pavilions with two wards of fourteen beds apiece in each, together with four single-bed wards in the block reserved for acute cases, and an operating theatre. The cruciform observation block contains twenty beds in single "wards," divided from each other by glass partitions. The centre of the cross is occupied by the nurses, who thus are able to observe all the patients, while the patients are protected from cross-infection. This plan has been applied only twice, we believe, before. The remodelled laundry is capable of dealing with 10,000 articles a week, and is entirely worked by electricity, so that the only person exposed to infection is the receiving officer. This elaborate machinery is the well-known type designed by Manlove, Alliot and Company, of Nottingham. The mortuary is furnished with glass screens. Two playing-sheds have been built for children to use in wet weather, and verandahs enable open-air treatment to be general. Steam heating on the central system has been installed by Messrs. E. H. May. The nurses' home contains twenty-four cubicles, with bathrooms on each floor and a general sitting-room on the ground floor. The medical superintendent, Dr. J. McGregor, hopes that if room can be found cases of measles may be admitted. So a forward policy and an ambitious building appear to be going hand in hand.

### THE SIGHTS INSPECTORS DO NOT SEE.

We are invited by a correspondent to return to the subject of the inspection of military hospitals. It will be recalled that on a previous occasion we discussed the suggestion that routine inspectors were sometimes a farce, since the inspectors often are content with the appearance of cleanliness, and are, on occasion, so chaperoned by the matron as to see little beyond what she deems advisable, and that little already prepared. "If an inspector," a correspondent writes, "is to be of any use, he should go disguised as a nurse or an orderly. Only so could he learn how much fuel is given out; only so could he take the temperature of the wards in the small hours of the morning; only so could he prove whether the allowance of food and so forth are really according to schedule. Do you know, sir, at ——, if a patient, as often happened, came in after 11 A.M., that is to say after the diet sheet had gone down, he could not have anything but milk until the third day after his admission? Under these conditions a healthy surgical case would be ferocious with hunger, and the weak ones become even weaker. Inspectors never came round when the patients were stealing each other's food, and when the thermometers were at freezing point." The hospital in question is in an exposed situation in one of the coolest parts of England. The suggestion is that if this had been recognised the inspectors would have been more thorough than they were. Would such women as former matrons make better inspectors?

### JOHN WESLEY AND THE HOSPITALS.

In John Wesley's journals there is more than one reference to the hospitals of the period. The diary entry for Saturday, December 6, 1740, for instance, is as follows: "4½ Drest, prayer! meditated, chocolate; 6 Matt. xiii., spinners, writ for society; 11 at Sister Aspernel's, conversed! 12 at home, conversed to many, spinners; 2 at Mr. Dolman's, dinner, conversed; 3.45 at the hospital; 4 at Guy's Hospital with S. Lincoln, Mr. Chandler, prayer, conversed! tea; 5.30 visited; 6 Long Lane; 8 at home, the women, prayer; 10." It should be remembered that a note of exclamation always indicates a special blessing in Wesley's diary. Mr. Wm. Wale, who has been through the eight volumes of the journal with a view to points of hospital interest, says that there is an entry recording a visit to St. Thomas's Hospital (then a near neighbour of Guy's) in 1741, when Wesley "visited a young man . . . who, in strong pain, was praising God continually. At the desire of many of the patients I spent a short time with them in exhortation and prayer." One gathers from what follows in this note that there was no chaplain at St. Thomas's at this time, or that Wesley was not impressed by the sufficiency of the arrangements for the visitation of the sick.

### LETTERS AND FRUIT.

A well-known London hospital chairman once declared that every appeal, or letter to a newspaper, should end with a sob or a smile. Here is an example of the latter from the provinces: "To the Editor, the *South Wales Daily News*. Sir,—Can you squeeze in the following items from to-day's receipts? 'My wife has got 10s. through selling surplus vegetables from my allotment. (I have got it now.) Herewith please find cheque eleven guineas (six guineas from my wife, five from self). It may interest you to know the £6 6s. is the proceeds of fruit grown in our small garden.' Who can say" (the letter concludes) "the appeal has not borne fruit after this?" The author of this letter is Mr. Leonard D. Rea, secretary of King Edward VII.'s Hospital, Cardiff. We hope that his letter will be as fruitful as the fruit which prompted it.

### POOR-LAW INFIRMARIES AND SOLDIERS' WIVES.

With reference to the note on this subject in last week's issue of The Hospital, page 502, it has transpired that the Bermondsey Board of Guardians have received a letter from the Local Government Board pointing out that they have been informed by the Army Council that only two cases of a soldier's wife, child, or other dependent had been notified to them as having received institutional treatment during the year ending April 30 last. They desire the observations of the Guardians on the matter. It appears that over 400 dependents of soldiers have been under treatment at the infirmary, and that at the commencement some ten cases were notified to the Army Council, but, as no acknowledgment of the information was received, the officer stopped sending the returns. It was his practice, however, to come to terms with the patients, and with their consent he deducted a few shillings a week from the allowances to cover the bare cost of the patient in the infirmary, the rest being devoted to keeping the children and the homes together. The Guardians considered this a very humane way of dealing with the sick poor, and have decided in the meantime that it shall be continued, and that no cases be notified to the Army Council; whilst the General Purposes Committee have decided to consider the reply that should be sent to the Local Government Board, who have expressed their dissatisfaction with the procedure adopted by the Board of Guardians.

### FARM COLONY FOR TUBERCULOUS SAILORS AND SOLDIERS.

A scheme with the above object projected, as already mentioned in our columns, by the National Association for the Prevention of Consumption, has now reached the stage of appeal for funds in the lay press. Five members of that body, including the chairman and vice-chairman and Sir William Osler, have written a letter appearing in the *Morning Post* of the 21st instant, stating that their Council is negotiating for the acquisition of a suitable property, and asking for £50,000 to lay it out as a National Farm Colony for the reception of sailors and soldiers broken down through tuberculosis. Donations will be gratefully acknowledged by Lord Glenconner at 20 Hanover Square, London, W. 1. The letter quotes the experience of Sir Robert Philip's similar institution near Edinburgh, which in four years turned out eighty-eight persons fit for agricultural employment. It will be seen from these last figures that no one institution of the kind, however large or efficient, is likely to meet the existing needs. We think, as we have said before, and as we repeat in the article "The After-Care of Consumptives," on p. 528 of this issue, that all such efforts need to be supplemented, or perhaps "complemented," by small farms run in connection with all public sanatoria on the land attached to them, catering for the sure market of their own constant requirements in the direction of food and transport, at any rate in the first place. All over Europe the public sanatoria are now almost filled with consumptive ex-combatants—Brehmer's institution is now called "Field-grey Görbersdorf"—so that a beginning in the right direction could be most quickly made by multiple action of the kind just recommended. And quick action is the way to avoid relapses into disease that may be irremediable.

### WHAT CAUSES MEASLES?

In these days, when committees are being appointed to inquire into all sorts of difficulties and problems, it has occurred to some that a Royal Commission on Measles would not be so useless as many Royal Commissions have proved to be. Measles has for centuries been a serious complaint, but no one appears to have discovered its cause. Now the Sanitary Committee of the Manchester Corporation has been thinking about the matter and adopted the following resolution: "That in view of the frequently recurring outbreaks of measles on an extensive scale throughout the country and the mortality among young children occasioned thereby, the Government should be urged to set aside a sum of money for research work into the causation of this disease and the best means of securing its prevention."

### METHODS AND MEASLES.

Is it possible to stamp out measles? This is the question raised by Dr. E. H. Snell in his annual report as medical officer of health for Coventry. He recalls that he has often previously urged that it is not necessary that children should suffer from measles, but that if they do the longer it can be postponed the less are the risks to the child. It is hoped, he says, that in time this fact may be appreciated by mothers. At present it is not, and numerous instances come to light where mothers designedly expose their other children to infection from a child suffering from measles "so that they can all get it over together." Medical men can be met with who think this course not unreasonable. That all children must have measles is as rooted a superstition as is the conviction that all dogs must have distemper. The eradication of this view holds out some hope of a lessening of the mortality from measles. The provision of a nurse in non-epidemic times may perhaps have some influence, and it is an experiment that might be made. Various methods of school closure or partial school closure have been tried, but all without success. It is probably an open question as to what effect administrative action can have in reducing the incidence and mortality of measles. Doubtless the fatality of the disease is influenced by the available medical and nursing services. In a city such as Coventry, where the bulk of the medical practice is of the contract variety, medical treatment should be available, and the assistance of the local nursing association might be further enlisted and officially recognised by the municipality.

### THE LATEST PHASE OF AFTER-CARE.

The opening by the Minister of Pensions of the Joseph and Jane Cowen Home for disabled sailors and soldiers at Benwell Grange, Newcastle, establishes on a more formal basis an institution which has already been at work for a year or more. After his return from France, in company with Sir Thomas Oliver and Sir Johnstone Wallace, Colonel Joseph Reed discussed the need for such a home in Newcastle with Miss Cowen, who gave him £2,500 to establish it. At first the men received were taught in various workshops, but it was found better to confine the training to the two suitable trades for which the local prospects are most promising—namely, boot and shoe making and electrical engineering. Of the 165 men who have been admitted about one-third has found employment, nearly one-third is still being trained, seventeen are under medical treatment, and eight have gone back to their original work. We only trust that the

Home will keep in touch with all who have left it, and preserve a record of their doings, successes and failures alike. Industrial after-care is no less important than therapeutic.

### MEDICAL HONOURS.

Lieut.-Colonel E. Goodall, one of a number of officers whose names have been brought to the notice of the Secretary of State for War for their valuable medical services, is head of the Metropolitan Hospital, Whitchurch, known as the Cardiff Mental Hospital until it was reorganised under the military authorities. Colonel Goodall, who is regarded as perhaps the greatest authority on lunacy in the Principality, was appointed medical superintendent of the Mental Hospital in 1907, having previously filled a similar position at the Carmarthenshire County Asylum. Captain H. A. Scholberg, bacteriological research expert at King Edward VII.'s Hospital, Cardiff, and the Cardiff Mental Hospital before the war, is mentioned for his services in aid of wounded soldiers at these institutions. Another officer who appears in the list is Lieut.-Colonel H. G. Cook, commanding the Welsh Hospital at Netley.

### THE GERM OF TYPHUS.

Early in the era of the germ theory of disease the objection was often brought against it that in many diseases certainly contagious no germ could be demonstrated, and for a time it was not easy to answer this objection; but one by one the germs of a very large number of the infectious diseases have been identified, until the argument in favour of the germ theory cannot be disputed by anyone acquainted with the facts of the case. Still, there are many diseases the germs of which have not yet been found. For many years unavailing researches for the germ of syphilis proceeded, but at last it was discovered. The germ causing typhus fever has eluded up to the present all attempts to discover it, but at last we learn that it has been found. Professor Kenso Futaki has been working in the Japanese Imperial Government Laboratory, and there he has carried on his investigations into typhus. He finds the germ to be a spirochæte, and he has given it the name of *Spirochæte exanthematotyphus*. The length of the name is partly due to the fact that the title of *Bacillus typhosus* has already been bestowed on the germ of typhoid fever. Professor Futaki has succeeded in demonstrating the organism in the bodies of human beings who have died from typhus, and he has also produced the disease in monkeys by injecting the germ, and in these infected monkeys he has found the germ again. The discovery of this organism is of no little importance, for typhus is endemic in some countries, and it is especially liable to appear in war-time, and in the course of the present conflict it has made its appearance in several parts of the continent of Europe, especially on the Eastern front. It is not improbable that the discovery of the germ producing the disease may be the first step in devising some method of inoculating against it, in the same way as we have already succeeded in reducing enormously the incidence of typhoid fever. Typhus is essentially a disease of dirt, and there is much evidence that it is conveyed from one patient to another by means of lice or bed-bugs. Even by nonspecific methods of prophylaxis the disease has almost disappeared from this country, and only occasionally is a case found.

### MALINGERER'S JAUNDICE.

The ways of the malingerer are passing strange. He utilises the most unlikely methods in the endeavour to hoodwink the examining surgeon, and in no department of life is the malingerer more active or more ingenious than when he wishes to evade military service. One of the latest tricks has recently been detected in Italy, where two soldiers were found to be suffering intensely from jaundice when they returned from leave, and yet two days previously they had been perfectly well. Many cases of jaundice have occurred during the war, some of them being catarrhal and some arising from other well-recognised sources, so that a case of jaundice, though artificially produced, might easily escape detection. In the case of the two soldiers already mentioned, the urine was deep orange in colour, but the usual tests for bile pigments failed completely. The urine, however, was found to contain picramic acid, and both of the soldiers confessed that they had taken picric acid. The dose taken appears to have been about twelve grains each day. There was much abdominal pain and nausea, and the urine and the conjunctivæ showed a yellow tint about twenty-four hours after the first dose. Instead of the typical pale clayey stools, the motions were yellow in colour. Now that the method has been exposed Army surgeons will be on the look out for these cases of artificial jaundice, and there will be little chance of their escaping detection.

### "GINGER" FOR CHELMSFORD.

Local authorities which hesitate to appoint health visitors are being gently approached by Whitehall. For instance, the Local Government Board has written to the Chelmsford Town Council stating that in view of the importance of taking every step possible to conserve infant life at the present time, the Board considers the appointment of a health visitor an urgent need in every district, and asks the Council to reconsider its decision not to appoint such a visitor.

### INFANT CONSULTATIONS.

Dr. Elizabeth C. McLaren's report to the Wolverhampton Corporation on "Infant Consultations" shows how necessary it is to have adequate staffs of health visitors. Certain mothers go eagerly and willingly to the clinic and faithfully carry out all instructions without the need of further help or correction, but others who are lazy or careless need continuous watching and visiting, and do not attend or try to improve without perpetual reminders from a health visitor, and at present the staff is not sufficient to ensure full supervision. She mentions that the proportion of breast-fed babies is only 60 per cent. This is below the usual average, but the apparently low figure is partly due to the widely divergent ages at which the infants attend. Many mothers appear for the first time when the baby is already eight or nine months old, and when artificial feeding has already begun, while others, who have lost their breast-milk early, arrive too late to make it possible to re-establish it. Breast-feeding would become almost universal and far more satisfactory if all mothers could spend the critical fortnight after the confinement entirely free from mental worry or physical strain. If it were possible to train steady elderly women as mothers' helps to take charge of the house during the period mentioned both mother and child would benefit permanently to the advantage of town and State. A certain amount of improvement could be effected if the maternity benefit were paid within ten days of the birth, at a time when it is most needed, instead of five or six weeks later, when the money is used for general purposes.

### NO ANXIETY ABOUT THE PLAGUE.

There were three cases of plague in Liverpool last year, but there is no need for panic. Dr. Hope, the medical officer of health, in his annual report, is pleasantly reassuring, stating that from the experience at other seaports the importation of foreign rags and their handling and sorting in rag stores lends opportunities for the spread of plague. The fact that one of the sufferers from plague worked in a rag-store caused complete inquiries to be made into the origin of all rags coming to this store; no foreign rags of any kind, however, had been received, the rags sorted at this store being all of local origin, and obtained from householders. Returns obtained from the Customs authorities regarding the arrivals of foreign rags gave no clue to the origin of this case of plague. It was deemed advisable, however, thoroughly to disinfect and cleanse both the rag-store concerned and also to purchase a quantity of loose rags, which were taken to the destructor and burnt. The outbreak confirms the experience of previous years that these localised outbreaks are all due to simple and specific importations of plague, which, once detected and promptly dealt with, can give little cause for anxiety. A prompt examination of all doubtful or suspected cases and a continuous and energetic campaign of rat-catching and rat-destruction both in the city, ships, and docks is, however, extremely important.

### THE COLOUR TREATMENT OF SHELL-SHOCK.

An attempt is to be made to test the value of colour in the treatment of shell-shock. Mr. Howard Kemp Prosser holds the theory that certain colours have a very beneficial influence in the treatment of nerve strain, and he has offered to decorate a hospital ward at his own expense with those colours which he thinks are likely to prove useful. The offer has been accepted, and the ward chosen is situated in Miss McCaul's Hospital for Officers in Welbeck Street, and the attempt has the approval of the military authorities. Mr. Prosser believes that in shell-shock the feeling that he is shut up within four walls is bad for the patient, and therefore he paints the ceiling the colour of the sky; the walls are painted yellow to produce the effect of sunlight; the curtains are sometimes yellow and sometimes blue, as blue has a soothing effect. For the most severe cases violet is said to be especially useful, but it is a colour that has to be employed with care, for it is a stimulant. The furniture must not be too obtrusive, and therefore it is painted yellow, but a little less intense than the colour of the walls. The floor is primrose and the woodwork is green, which is the colour of early spring and hope. The crockery is blue. We await with interest the result of this experiment, and we are glad that the authorities have sanctioned it, for there can be no doubt that the colours of a room have a great effect on its inhabitants, and the question of colours is not sufficiently considered from the hygienic point of view.

### ADDISON'S DISEASE IN A RECRUIT.

A case which has caused no little trouble has recently engaged the attention of the authorities. The Lower Board classified the man as C1, although he had a certificate stating that he was suffering from Addison's disease. The Special Medical Board then considered the case, and three medical men gave evidence that the recruit was suffering from Addison's disease, but the Board confirmed the judgment of the Lower Board. When the case came before the Spring Gardens Tribunal it was resolved that though the Tribunal was satisfied that the Special Board had exercised the greatest care in the examination of the patient, as there was some conflict of medical opinion he should be granted conditional exemption, as it had been stated that the man was really in danger of sudden death. It is difficult for the public to understand that there may be a real difference of opinion as to the existence of such a disease in a patient; it is too often thought that it must be easy to say whether he is suffering from it or not. While it is simple in most cases to recognise an advanced case of Addison's disease, in the milder examples of the disease and in the earlier stages there may well be a conflict of testimony. The case certainly should be watched, for if it is an example of Addison's disease it will not be long before the symptoms or a fatal result clinches the diagnosis.

### AN ENDOWED BED TO THE MEMORY OF A COMMERCIAL TRAVELLER.

The Hospital is always pleased to record instances in which the memories of worthy men are perpetuated. A good example comes from Leicester Royal Infirmary, where Mrs. M. A. Shipley, of Melton Mowbray, has sent a cheque for £1,000 to endow a bed to the memory of her brother, the late Mr. John Platts, who, in the terms of the inscription which is to be placed above the bed, "was for many years closely associated with medical work in this neighbourhood." For close on half a century Mr. Platts represented the interests of the well-known firm of druggists, Messrs. Southall Bros. and Barclay, Ltd., and so successful was his selection of his customers that it is on record that in several years he did not make a single bad debt. He possessed a singularly amiable and kindly disposition, which at once commended him to his customers and led to many friendships formed in the course of his long service, friendships which were never broken and which were continually "kept green" by many little reminders in the intervals between his journeys, by newspapers, press cuttings, and postcards on any subjects in which he knew his correspondents to be interested. His sudden death at Oxford while on his regular journey was received with widespread regret. His name will be honoured and long remembered. By his firm he was held in the highest esteem, and he rose to a position of prominence from which he was able to accumulate a fortune of about £20,000. He enjoyed in the fullest degree the personal friendship of Sir Thomas Barclay, the head of the firm. When, therefore, the question of a memorial was suggested to Sir Thomas Barclay, it was only natural to find him submitting the idea to Mrs. Shipley, to whom Mr. Platts' fortune passed, with a cordial recommendation that the endowment of a bed in Leicester's county hospital was appropriate and most useful—a recommendation happily adopted by Mrs. Shipley in the incident now recorded.

### WOMEN PORTERS IN INSTITUTIONS.

The scarcity of men is having the result of compelling most managers of institutions to rely upon women. A typical case in point is the Lambeth Infirmary. Male porters are impossible to obtain, but four are wanted. The superintendent, therefore, is getting six women to act as porters, at a salary of 28s. a week with some emoluments worth 2s. 3d. a week. The men used to get £2 and emoluments worth 4s. In his report to the Board the superintendent says that the amount of female labour in the infirmary has grown to such an extent that further supervision is deemed necessary by the matron, who has engaged a supervisor at a salary of £2 a week, with lunch and tea. The matron thinks the increased cost will in all probability be met by a decrease in the number of women who will be required owing to better supervision. At the present time there are as many as sixty-two women employed as scrubbers, porters, and orderlies.

### THE THIRD LONDON "GAZETTE."

We are pleased to see that the September number of the *Gazette* of the 3rd London General Hospital, Wandsworth, reminds its readers that the late editor, Corporal Ward Muir, has published that agreeable volume "Observations of an Orderly" through Messrs. Simpkin, Marshall, price 2s. 6d. Probably most readers of The Hospital are acquainted with the book, which possesses its value as showing the ability of the editor, which made the *Gazette's* reputation. In his own pages his personality is fully revealed, and its impress the outside world can now see was upon every page of the paper which he edited. The fly is still a suitable topic for summer conversation, and it is with a light but competent touch that Sergeant H. J. Gilly describes the machinations of the creature as a disseminator of bacteria. Drawings of the "Brothers Cocci" accompany the text and make the various members of the family distinguishable. It all makes capital reading, and we hope that Sergeant Gilly will return to the subject in future articles. Miss H. M. Nightingale has three good and two poor verses on "Russia," but the present number is enough to show that the fund of originality among the contributors is not exhausted.

### THE HELMETED NURSE.

One result of the bombing of the hospitals behind the British and French lines in France by the Germans is said to be the issue of steel helmets to the nursing staff. It is clear, at all events, that by this time the problem of protecting hospitals, staffs, and patients by something else than a red cross painted on the roofs will have been attempted; and the helmet, like the gas-mask, may have been found as useful to the nurse as to the soldier. But it is a horrible thought that one more barrier between the combatant and the non-combatant has been apparently broken down.

### THIS WEEK'S DRUG MARKET.

A fair volume of business has been done since last report appeared, but the most important part of the buying in these days is on behalf of one or other of the Governments engaged in the war. The amount of business done in the ordinary way for home account is, however, by no means insignificant in spite of the difficult conditions of the market. The prices of salicylic acid and salicylate of soda are not quite so firmly maintained, and drugs of British manufacture are offered rather more freely. Podophyllin is scarcer and dearer. The scarcity of phenacetin is not quite so pronounced, and the price tendency is in a downward direction. Pepsin is rather scarce, and the price tends upwards. The value of camphor is firmly maintained. There is still a big demand for glycerophosphates. Sodium bicarbonate is dearer. Still higher prices are asked for saccharin, but it is anticipated that supplies will shortly be rather more plentiful. Clove oil is again much dearer owing to a further advance in the value of cloves. The price of American peppermint oil is substantially higher. There is no relief of the scarcity of cream of tartar.

### TO OUR READERS.

Contributions are specially invited from any of the readers of The Hospital. They should deal with topical subjects and news or incidents illustrating any department of hospital work, its progress or difficulties. They must be authenticated for the information of the Editor only. The minimum payment if published is 5s. There is no hard-and-fast rule as to space, but paragraphs of about twenty lines in length are preferred.

